# Characterization of the probiotic properties of *Lacticaseibacillus rhamnosus* LR6 isolated from the vaginas of healthy Korean women against vaginal pathogens

**DOI:** 10.3389/fmicb.2023.1308293

**Published:** 2023-11-30

**Authors:** Yusook Chung, Seung Beom Kang, Dooheon Son, Ji Young Lee, Myung Jun Chung, Sanghyun Lim

**Affiliations:** ^1^R&D Center, Cell Biotech, Co., Ltd., Gimpo-si, Republic of Korea; ^2^Department of Obstetrics and Gynecology, Research Institute of Medical Science, Konkuk University School of Medicine, Seoul, Republic of Korea

**Keywords:** *Lacticaseibacillus rhamnosus*, vaginal probiotics, vaginal microbiome, bacterial vaginosis, vaginal candidiasis, bacteriocin, adhesion

## Abstract

The human microbiome exhibits intricate populations across the body, with the vaginal tract serving as an ecosystem characterized by the prevalence of the genus *Lactobacillus*. Disruptions in the vaginal microbiota, which are frequently linked to variables such as sexual activity, hormonal fluctuations, and excessive use of antibiotics, can result in vaginal dysbiosis and the development of diseases such as bacterial vaginosis (BV) and candidiasis. *Lactobacillus* species, owing to their capacity to create an acidic environment through the production of lactic acid, have a key function within this complex microbial community: they inhibit the growth of harmful microorganisms. This study aimed to investigate the genomic characteristics of *L. rhamnosus* LR6, a newly discovered strain isolated from the vaginal microbiota of 20 healthy women to assess its potential as a vaginal probiotic. We performed a comparative investigation of the genetic traits of *L. rhamnosus* using 45 publicly available genomes from various sources. We evaluated the genetic characteristics related to carbohydrate utilization, adhesion to host cells, and the presence of bacteriocin clusters. A comprehensive study was conducted by integrating *in silico* evaluations with experimental techniques to authenticate the physiological characteristics of strain LR6. We further used a rat model to assess the impact of *L. rhamnosus* LR6 administration on the changes in the gastrointestinal tract and the vaginal microbiome. The assessments revealed a significantly high inhibitory activity against pathogens, enhanced adherence to host cells, and high lactic acid production. Rat experiments revealed changes in both the fecal and vaginal microbiota; in treated rats, *Firmicutes* increased in both; *Lactobacillaceae* increased in the fecal samples; and *Enterobacteriaceae* decreased but *Enterococcaceae*, *Streptococcaceae*, and *Morganellaceae* increased in the vaginal samples. The study results provide evidence of the genetic characteristics and probiotic properties of LR6, and suggest that oral administration of *L. rhamnosus* LR6 can alter both gut and vaginal microbiome. Collectively, these findings establish *L. rhamnosus* LR6 as a highly promising candidate for improving vaginal health.

## 1 Introduction

The human microbiome presents complex patterns throughout the body; the vaginal tract is a particular ecosystem in which one genus, *Lactobacillus*, is predominant (Chee et al., 2020). Because of the presence of these lactic acid-producing bacteria, healthy vaginal tracts are regularly acidic, and this acidic environment can inhibit the overgrowth of potentially harmful bacteria. Although the exact reason for the disruption of the vaginal microbiota is unknown, dysbiosis is strongly associated with bacterial vaginosis (BV) caused by sexual activity, hormonal changes, or overuse of antibiotics (Abbe and Mitchell, 2023). The most prevalent bacteria that commonly cause BV include *Gadnerella vaginalis*, *Escherichia coli*, and several *Prevotella* species (Srinivasan et al., 2012). Similar to BV, candidiasis, is also a major cause of infection caused by *Candida* spp. (Pappas et al., 2018).

Several drugs, including metronidazole or clindamycin for BV, and fluconazole and clotrimazole for *Candidal vaginitis*, are efficient therapies for vaginal infections. However, these treatments can exacerbate vaginal dysbiosis, which is already present in vaginal infections, potentially disrupting the delicate balance of the vaginal microbiota by affecting beneficial microorganisms (Bradshaw et al., 2006; Schuyler et al., 2016). In contrast, probiotics can promote the recovery process by restoring the vaginal microbiota’s environment (Liu et al., 2023). *Lactobacilli*, recognized for their capacity to maintain an acidic microenvironment through lactic acid production, play an essential role in this intricate microbial community by impeding the growth of detrimental microbes (Wu et al., 2022).

*Lactobacillus rhamnosus*, a species thoroughly studied within the *Lactobacillus* genus, has been isolated in many environments such as the human gastrointestinal tract and dairy products. This species is widely recognized for its potential to modulate the gut microbiota and enhance the immune system (Vélez et al., 2010; Martín et al., 2019). Therefore, it is frequently used as a probiotic supplement and has been integrated into many food products (Westerik et al., 2018). Not just in human gut system, it also has a great potential for probiotics for promoting vaginal health owing to antimicrobial characteristics, capacity to adhere to vaginal epithelial cells, and ability to produce hydrogen peroxide (Petrova et al., 2021). Increasing attention toward the therapeutic use of probiotics for improving vaginal health has inspired researchers to explore novel strains that exhibit enhanced beneficial properties (Liu et al., 2023).

This study aimed to explore the genomic attributes of *L. rhamnosus*, LR6, a newly isolated strain from the vaginal microbiota of a healthy female. We investigated the genetic characteristics of LR6, obtaining insights into its potential vaginal probiotic properties to adapt to the vaginal environment, by conducting a comparative analysis of its genomic properties with publicly accessible genomes of *L. rhamnosus* obtained from diverse origins. The inclusion of this substantial number of genomes serves to enhance the robustness of the comparative genomic analysis, facilitating a deeper understanding of the species’ genetic variation and potential probiotic capabilities. In addition, we revalidated the physiological traits of the strains inferred from their genetic features by utilizing various vaginal pathogens and host cells, not only through *in silico* analysis but also through experimental approaches.

## 2 Materials and methods

### 2.1 Isolation of *Lacticaseibacillus* LR6 strain from the vaginal tracts of healthy women

Between November and December 2022, vaginal swab samples from 20 healthy Korean women were used to obtain the clinically isolated *L. rhamnosus* strain LR6. The subjects ranged in age from 19 to 50 years, had regular menstrual cycles, and had no vaginitis or cervicitis symptoms (Nugent score of 1–3 points for vaginal discharge). The study protocol was approved by the Institutional Review Board of the Konkuk University Medical Center (KUMC 2022-04-030). The vagina swab kit (Noblebio, South Korea) was used as a vaginal discharge sampling kit and collected through internal examination at the Department of Obstetrics and Gynecology using a vaginal swab kit (Noblebio, South Korea). The swabs were streaked on de Man, Rogosa, and Sharpe (MRS) agar plates (Difco, Detroit, MI, USA) supplemented with 0.01% sodium azide and incubated at 37°C under aerobic conditions for 24 h. Using the single colonies, colony PCR was performed using the universal 16S rDNA sequences primers 27F (5’-AGAGTTTGATCCTGGCTCAG-3’) and 1492R (5’-GGTTACCTTGTTAGACTT-3’).

### 2.2 DNA extraction and whole-genome sequencing

DNA from the bacterial resuspension of *L. rhamnosus* LR6 was with DNA Link (DNA Link Inc., Seoul, Korea) using the QIAGEN MagAttract HMW DNA kit (QIAGEN, Hilden, Germany), following the manufacturer’s instructions. Using Megaruptor v3, we generated 10 kb fragments by shearing genomic DNA according to the manufacturer’s recommended protocol. An AMpureXP bead purification system was used to remove small fragments. A total of 500 ng of each sample was used as input for library preparation. The SMRTbell library was constructed using the SMRTbell Express Template Preparation Kit v2.0 (101-685-400). Samples were pooled according to the volumes provided by the Microbial Multiplexing Calculator, and small fragments of less than 3 kb were removed using the AMpureXP bead purification system for the large-insert library. After the sequencing primer was annealed to the SMRTbell template, DNA polymerase was bound to the complex (Sequel II Binding kit 3.2). The complex was purified using AMPure to remove the excess primers and polymerase prior to sequencing. Library preparation and sequencing were performed using DNA Link (DNA Link Inc., Seoul, Korea). The SMRTbell library was sequenced using SMRT cells (Pacific Biosciences) with the Sequel II Sequencing Kit v2.0, and 1 × 15 h movies were captured for each SMRT Cell 8M using the Sequel II (Pacific Biosciences) sequencing platform.

### 2.3 Comparative genomics of *L. rhamnosus*

The 45 publicly available whole-genome sequences (status: complete, 2023.06) of *L. rhamnosus* were retrieved from the NCBI database. These genomes, including that of the newly isolated LR6 strain, were used for comparative genomic analysis. All genomes were converted to GFF format using the Roary pipeline (Page et al., 2015), and orthologs were clustered into the core genome, pan-genome, and gene sets with accessory and unique genes. Clustered gene sets and statistical information were subjected to the Pagoo pipeline, R package for pan-genome analysis (Ferrés and Iraola, 2021). Core gene sets were aligned using the Roary pipeline, and the Fasttree program was used to generate a phylogenetic tree. The constructed phylogenetic tree was visualized using the iTOL web tool (Letunic and Bork, 2021). The 45 genome sequences were used to annotate carbohydrate-active enzymes and substrates (CAZymes) using the dbCAN3 standalone version in the HMMER and dbCAN3 CAZyme databases (Zheng et al., 2023). Automatically annotated CAZyme sequences were reclassified into five classes: carbohydrate-binding protein module families (CBM), carbohydrate esterases (CEs), glycoside hydrolases (GHs), glycosyltransferases (GTs), and polysaccharide lyases (PLs). To observe the genetic distribution of LR6 mucosal adhesion genes associated with host interaction, spaCBA-strC1 (spaA:LGG_00442, spaB:LGG_00443, spaC:LGG_00444, srtC1:LGG_00441) spaFDE-strC2 (spaD:LGG_02370, spaE:LGG_02371, spaF:LGG_02372,srtC2:LGG_02369), mucus-binding factor (MBF) with Pfam-MucBP domain repeats (LGG_02337), adhesion modulator MabA (LGG_01865), and lectin-like protein-coding gene (LGR1_llp1) sequences were retrieved and used as query for blast analysis. Only genes with sequence similarity and coverage of >90% were used. Bacteriocin clusters were identified using BAGEL 4 standalone version (Van Heel et al., 2018). According to the area of interest, core and accessory genes were visualized using the gggenes package (ver. 0.5.1).

### 2.4 Growth conditions of bacterial strains

Three strains of *L. rhamnosus* were used in this study: one newly isolated from the human vagina (*L. rhamnosus* strain LR6) and two control strains (*L. rhamnosus* ATCC 7469T and LGG). All *L. rhamnosus* strains were incubated in de MRS broth for 18 h at 37°C. Three pathogens were used to determine the inhibitory effects of lactic acid bacteria on BV. *Candida albicans* ATCC 18804 and *Escherichia coli* ATCC 11775 were cultivated aerobically in yeast extract–peptone–dextrose (YPD) and Luria–Bertani (LB) broth media, and *Gardnerella vaginalis* ATCC 14018 was cultured in trypticase soy broth (TSB) with 2% of vitamin K1-hemin solution at 37°C under anaerobic conditions. To compare the potential survival in an acidic tolerance under anaerobic conditions, *L. rhamnosus* were cultured in pH3, 4, 5, and 6 in MRS media for 18h at 37°C under the aearobic conditions, and optical density (OD) at 600nm were measured.

### 2.5 Antibacterial and antifungal activities

To assess the antimicrobial activity of the three *L. rhamnosus* strains (LR6, L7469T, and LGG), *C. albicans* was inoculated into YPD media, *G. vaginalis* into TSB media, and *E. coli* into LB media. The supernatant from each *L. rhamnosus* strain was obtained by filtering bacterial cells (0.2 μm) after an 18-hour culture in MRS liquid medium. Following 18 hours of co-incubation with the respective pathogens, the inhibitory effect of the LR6, L7469T, and LGG supernatants was evaluated by measuring the optical density (OD) at 600 nm of the pathogen cultures.

### 2.6 Co-aggregation assay

Co-aggregation assay of *Lactobacillus* and vaginal pathogens was performed as previously described (Reuben et al., 2019). The culture media and conditions for *L. rhamnosus* LR6, *C. albicans*, *G. vaginalis*, and *E. coli* were the same as described in section “2.4. Growth conditions of bacterial strains” Cultures of *L. rhamnosus* LR6 and the pathogens were collected, washed with phosphate-buffered saline (PBS) twice, and each diluted to a standardized OD of 1.0 at 600 nm using PBS. A 2ml suspension of *L. rhamnosus* LR6 at OD 1.0 and a 2ml suspension of each pathogen at OD 1.0 were then co-incubated in round-bottom tubes at 37°C for 4 hours. Similarly, 4ml suspensions of *L. rhamnosus* and each of the pathogens were prepared individually under the same conditions. The OD of each single microbe suspension (ODLR and ODpathogen) and mixed suspension (ODmix) was measured at 600 nm, and the co-aggregation ratio was calculated as follows:

$$C\,o\,a\,g\,g\,r\,e\,a\,t\,i\,o\,n\%=\left[1-\frac{2\,\left(O\,D\,m\,i\,x\right)}{O\,D\,L\,R+O\,D\,p\,a\,t\,h\,o\,g\,e\,n}\right]\times 100$$

### 2.7 HeLa cell adhesion assay

HeLa cells used in this study were purchased from the Korean Cell Line Bank and cultivated on Park Memorial Institute 1640(RPMI 1640, Gibco, USA) supplemented with 10% fetal bovine serum(FBS, Gibco, USA) at 37°C for 2 days in 5% CO2. The HeLa cells were diluted to a concentration of 5 × 10^4^ cells per well and incubated at 37°C and 5% CO_2_ for 48 h. When the cells reached 80% confluence, dead cells were removed using PBS and distilled water (DW). Subsequently, each *L. rhamnosus* strain was inoculated at a concentration of 2 × 10^8^ colony-forming units per milliliter (CFU/mL) and incubated for 1 h at 37 °C. Each well was washed with pre-PBS and DW and treated with trypsin and ethylenediaminetetraacetic acid (EDTA). Cells containing *L. rhamnosus* were quantified by colony counting on the MRS agar plates.

### 2.8 Measurement of metabolic acid-producing ability

The *L. rhamnosus* strains were cultured in MRS broth to confirm their acid-producing abilities. After centrifugation 15 m, 37 °C, 4000rpm, the supernatant was collected and sterilized by membrane filtration (0.2 μm). The concentrations of acetic, citric, and lactic acids were measured using high-performance liquid chromatography equipped with UV-DAD (HPLC Infinity 1290, Agilent Technologies). The separation of targeted metabolic acids was conducted on the reversed-phase analytical column CAPCELL PAK C18 MG (4.6 mm × 250 mm, 5 μm, Shiseido) maintained at 30°C. The binary mobile phase contained both A and B was 10 mM potassium dihydrogen phosphate (KH2PO4, pH 2.4 adjusted with phosphoric acid) and acetonitrile. The gradient of mobile phases was as follows: 0 min, A 100% B 0%; 7.5 min, A 100% B %; 8 min, A 85% B 15%; 16 min, A 85% B 15%; 17 min, A 0% B 100%; 18 min, A 0% B 100%; 19 min, A 100% B 0%; 20 min, A 100% B 0% for re-equilibrium. The flow rate was 1mL/min, UV wavelength was 210 nm and analytical time was 20 min per sample. An aliquot of 10 μL was injected and each sample was analyzed in triplicate.

### 2.9 Measurement of hydrogen peroxide producing ability

The hydrogen peroxide generating ability of the isolated strains was measured by streaking the strains on MRS–TMB agar (TMB: 3,3′,5,5′-tetramethylbenzidine), followed by their incubation at 37 °C for 48 h under anaerobic conditions. The plates were exposed to air for 30 min. Hydrogen peroxide-producing strains were identified by the conversion of white colonies to blue colonies owing to TMB oxidation.

### 2.10 Fecal and vaginal microbiota

Female 6-week-old Sprague-Dawley rats (*n* = 12) were purchased from Orient Bio. The objective was to assess the impact of *L. rhamnosus* LR6 administration on the changes in the microbiome of the gastrointestinal tract and vagina. The rats were housed in an environmentally controlled room (24 ± 2 °C, 40–60% relative humidity) under a 12 h light/dark cycle, and had free access to food and water during a 1 week adaptation period. The animal use protocol was reviewed and approved by the Institutional Animal Care and Use Committee board in the CellBiotech (IACUC, approval No: CBT-2023-09) based on guidance of the Association for Assessment and Accreditation of Laboratory Animal Care (AAALAC). The rats were randomly assigned to two groups (*n* = 6 per group). The negative control group was administered PBS, and the experimental group was administered *L. rhamnosus* LR6 (3.82 × 10^9^ CFU/rat) for 2 weeks, daily basis for two weeks. The same volume of PBS (pH 7.4) was used as the negative control. Fecal pellets were collected by inducing defecation through gentle abdominal massage of the mice. Following this, 100 μL of sterile phosphate-buffered saline (PBS) was used to lavage the vaginal lumen, which allowed for the collection of vaginal content samples. Fecal samples were collected on days 0 and 14 and vaginal swabs were collected on day 14.

### 2.11 Microbiome analysis

The fecal and vaginal samples were vigorously agitated into to buffer to dislodge the cells. The SPINeasy DNA Pro kit for Soil (MP Biochemicals, Santa Ana, CA, USA) was used to extract DNA from the mouse vagina after it washed with PBS. The Illumina 16S Metagenomic Sequencing Library Preparation guide was followed during the processing of the extracted DNA to construct a sequencing library. We used a forward primer in the V4 region (CCA GCMGCC GCG GTA ATW C) and a reverse primer in the V5 region (CC GTC AAT TYY TTT RAG TTT) to target the V4–V5 region of the bacterial 16S rRNA gene for 16S rRNA gene sequencing. The Nextera XT v2 Index Kit was then employed to perform index PCR in order to generate a library. Using the MiSeq reagent kit V2, the NGS process involves sequencing 250-bp paired-end reads on the MiSeq platform (Illumina, San Diego, CA, USA). Qiime2 (QIIME2, v2023.5, viewed on June 26, 2023)^1^ was used for data processing on the FASTQ file, and DADA2 was applied for sequence quality verification.

### 2.12 Statistical analysis

Unpaired two-tailed *t*-tests for single comparisons or Wilcoxon rank-sum tests were used to assess the significance of differences in *in vitro* experimental data. A value of *P* < 0.05 was considered to be statistically significant.

## 3 Results

### 3.1 Genomic characteristics of newly isolated vaginal Lactobacilli

**FIGURE 1:**
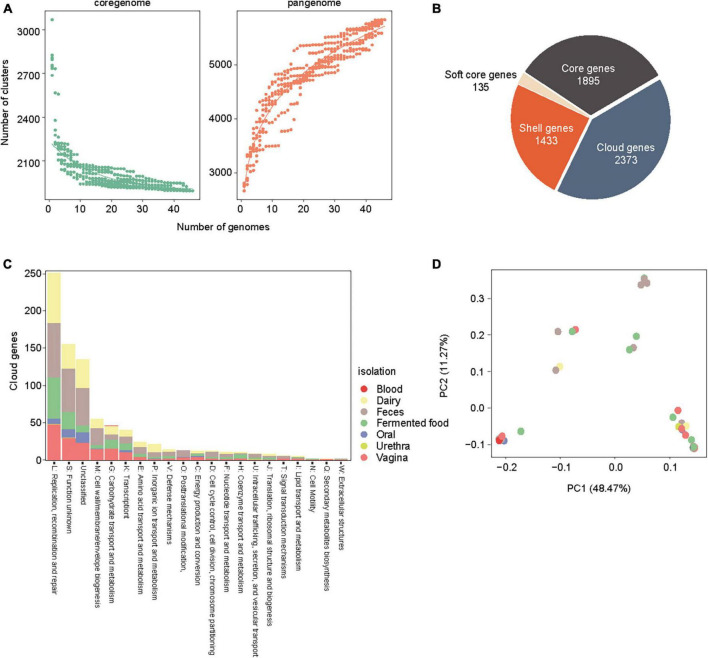
Core genome and pan genome analysis of the 45 *L.rhamnosus* strains. **(A)** Accumulation curves of core genome and pan genome of *L. rhamnosus* strains. **(B)** The number of core genes, cloud genes, shell genes and soft core genes are presented in pie chart. **(C)** COG category abundances in cloud genes of *L. rhamnosus* strains. Isolation sites were colored in the bar of COG category. **(D)** A principal components analysis generated from 45 *L. rhamnosus* genomes. Isolation sites were colored in the spots.

To elucidate the genomic features of the recently isolated strain of *L. rhamnosus* LR6 from the vaginal microbiota of healthy females, a comparative genomics analysis was conducted. This analysis involved a comparison of the genomic sequences of the newly isolated strain with those of 45 publicly accessible genomes of *L. rhamnosus*. This study included 45 strains of *L. rhamnosus*, which were obtained from various sources; these sources were feces (18 strains), fermented products (12 strains), dairy products (2 strains), the vagina (6 strains), urethra (1 strain), blood (1 strain), oral (2 strains), and unknown sources (3 strains). The genome sizes of these strains ranged from 2.88 Mb to 3.25 Mb, as shown in Table 1. Based on a study of the core and pan-genome, 1895 genes were identified and classified as the core gene set. Additionally, 1433 and 2373 out of the 5836 genes were estimated to belong to the shell and cloud gene categories, respectively (Figures 1A, B). To examine the evolutionary relationships among the sources of origin, 2373 cloud genes were annotated using the Clusters of Orthologous Genes (COG) database and subsequently classified based on their isolation sources (Figure 1C). Analysis of the 20 COG categories indicated that the functions of replication, recombination, and repair (L) were the most prevalent among the cloud genes derived from various sources, such as dairy, feces, fermented food, and oral and vaginal sources. Notably, the specific function of cloud genes originating from a second source remains unidentified, although they potentially play a role in the development of distinct genetic traits. In the case of the strains of vaginal origin, 29 genes were identified and annotated for carbohydrate transport and metabolism (G) and cell wall/membrane/envelope biogenesis (M), especially phosphotransferas (PTS) systems for various carbon sources and glycosyltransferase-like families. The principal component analysis (PCA) thoroughly differentiated genomes obtained from the vagina into three distinct phylogroups. However, no discernible pattern was identified based on the source of isolation, as shown in Figure 1D.

**TABLE 1:** Forty five strains of *L. rhamnosus* used in this study.

Strain	Accession	Size (Mb)	GC%	CDS	Origin
*Lacticaseibacillus rhamnosus* LOCK900	GCA_000418475.1	2.88	46.80	2578	Feces
*Lacticaseibacillus rhamnosus* Pen	GCA_002076955.1	2.88	46.80	2573	Feces
*Lacticaseibacillus rhamnosus* VHProbi M14	GCA_024610975.1	2.90	46.70	2628	Fermented food
*Lacticaseibacillus rhamnosus* LV108	GCA_013167115.1	2.92	46.80	2630	Feces
*Lacticaseibacillus rhamnosus* B6	GCA_016599675.2	2.92	46.80	2628	Fermented food
*Lacticaseibacillus rhamnosus* AS	GCA_018286375.1	2.94	46.80	2583	Fermented food
*Lacticaseibacillus rhamnosus* CE1	GCA_018141205.1	2.94	46.80	2587	Fermented food
*Lacticaseibacillus rhamnosus* Strain 1.032	GCA_006151905.1	2.94	46.70	2622	Fermented food
*Lacticaseibacillus rhamnosus* ATCC 8530	GCA_000233755.1	2.96	46.80	2621	Feces
*Lacticaseibacillus rhamnosus* CLK 101	GCA_024442295.1	2.96	46.64	2635	Fermented food
*Lacticaseibacillus rhamnosus* GR-1	GCA_024665595.1	2.97	46.64	2638	Urethra
*Lacticaseibacillus rhamnosus* LR6	in this study	2.97	46.74	2782	Vagina
*Lacticaseibacillus rhamnosus* LR5	GCA_002286235.1	2.97	46.70	2659	Feces
*Lacticaseibacillus rhamnosus* NCTC13764	GCA_900636965.1	2.99	46.80	2654	Unknown
*Lacticaseibacillus rhamnosus* BIO5326	GCA_009720565.1	2.99	46.80	2655	Feces
*Lacticaseibacillus rhamnosus* SCT101060	GCA_002960215.1	2.99	46.80	2648	Feces
*Lacticaseibacillus rhamnosus* JCM1553	GCA_003433395.1	2.99	46.80	2641	Unknown
*Lacticaseibacillus rhamnosus* A5	GCA_029543065.1	2.99	46.80	2617	Fermented food
*Lacticaseibacillus rhamnosus* LOCK908	GCA_000418495.1	2.99	46.80	2665	Feces
*Lacticaseibacillus rhamnosus* CAU 1365	GCA_019967935.1	2.99	46.80	2667	Fermented food
*Lacticaseibacillus rhamnosus* NCTC13710	GCA_900636875.1	2.99	46.80	2651	Unknown
*Lacticaseibacillus rhamnosus* VHProbi Y39	GCA_022220485.1	2.99	46.80	2655	Feces
*Lacticaseibacillus rhamnosus* X253	GCA_018228745.1	2.99	46.80	2649	Fermented food
*Lacticaseibacillus rhamnosus* P118	GCA_023913535.1	2.99	46.80	2648	Fermented food
*Lacticaseibacillus rhamnosus* VSI33	GCA_029010255.1	2.99	46.80	2649	Vagina
*Lacticaseibacillus rhamnosus* PMC203	GCA_020826335.1	2.99	46.66	2647	Vagina
*Lacticaseibacillus rhamnosus* HN067	GCA_024397415.1	3.00	46.75	2666	Feces
*Lacticaseibacillus rhamnosus* ATCC 53103	GCA_000011045.1	3.01	46.70	2681	Feces
*Lacticaseibacillus rhamnosus* BIO6870	GCA_008831425.1	3.01	46.70	2691	Feces
*Lacticaseibacillus rhamnosus* GG	GCA_028475085.1	3.01	46.70	2699	Feces
*Lacticaseibacillus rhamnosus* LDTM7511	GCA_017795605.1	3.01	46.70	2665	Feces
*Lacticaseibacillus rhamnosus* LR-B1	GCA_004010975.1	3.01	46.70	2677	Blood
*Lacticaseibacillus rhamnosus* MGYG-HGUT-01293	GCA_902381635.1	3.01	46.70	2690	Feces
*Lacticaseibacillus rhamnosus* DSM 14870	GCA_002287945.1	3.01	46.70	2697	Vagina
*Lacticaseibacillus rhamnosus* DM065	GCA_024053515.2	3.02	46.59	2702	Oral
*Lacticaseibacillus rhamnosus* VHProbi F20	GCA_026427555.1	3.02	46.69	2717	Feces
*Lacticaseibacillus rhamnosus* BPL5	GCA_900070175.1	3.02	46.70	2704	Vagina
*Lacticaseibacillus rhamnosus* DM054	GCA_024158105.2	3.03	46.69	2704	Oral
*Lacticaseibacillus rhamnosus* Lc 705	GCA_000026525.1	3.03	46.63	2648	Dairy
*Lacticaseibacillus rhamnosus* 4B15	GCA_002158925.1	3.05	46.70	2734	Feces
*Lacticaseibacillus rhamnosus* VSI43	GCA_029011275.1	3.05	46.73	2692	Vagina
*Lacticaseibacillus rhamnosus* TK-F8B	GCA_015377485.1	3.06	46.65	2728	Fermented food
*Lacticaseibacillus rhamnosus* hsryfm 1301	GCA_008727835.1	3.07	46.75	2769	Feces
*Lacticaseibacillus rhamnosus* BFE5264	GCA_001988935.1	3.11	46.76	2785	Dairy
*Lacticaseibacillus rhamnosus* KF7	GCA_016653515.1	3.25	46.64	2902	Fermented food

### 3.2 Genes associated with carbohydrate utilization enzymes and mucosal adhesion

**FIGURE 2:**
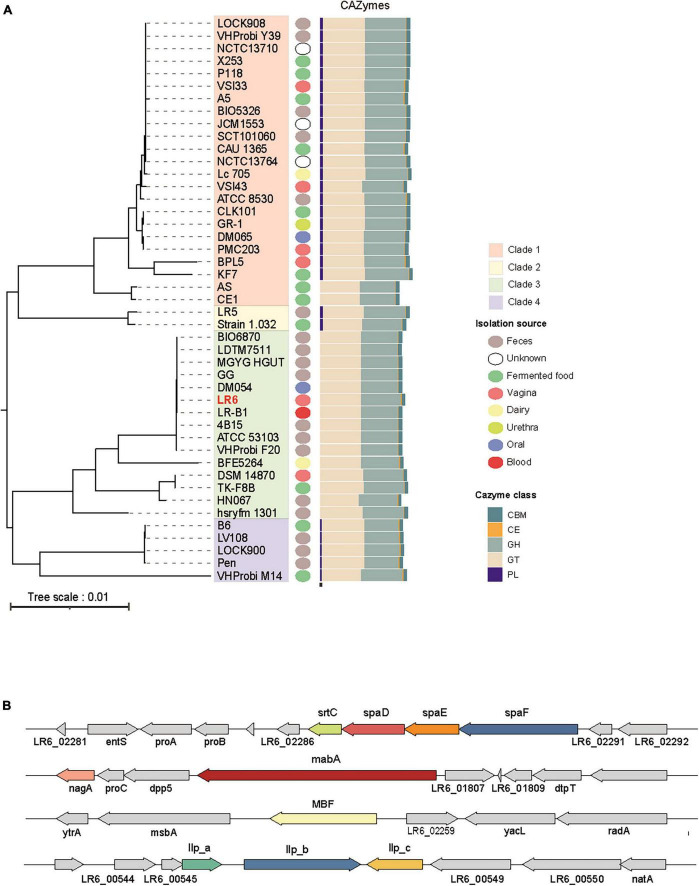
Phlyogenomic analysis of the 45 *L. rhamnosus* strains. **(A)** RAxML tree of 45 *L. rhamnosus* constructed from core-genome alignment. Branches are colored by four phylogroups. Each colored circle denoted isolation source and five classes of CAZymes utilization profiles are shown. **(B)** Mucosal adhesion genes associated with host interaction in *L. rhamnosus* LR6 were annotated through the blast search and four genomic regions are shown.

The concatenated and aligned core genes were used to construct a phylogenetic tree of the 45 genomes, and four clades were identified (Figure 2). To explore the genetic basis of the four clades, the distribution of genes encoding carbohydrate utilization enzymes was analyzed using the CAZymes database and dbCAN3. Of the total 6759 genes, there were 836 CBM, 135 CEs, 3197 GHs, 2540 GTs, and 51 PLs. The CAZyme profiles showed that clades 1, 2, and 4 commonly possessed all types of CAZyme classes (CBM, CE, GH, GT, and PL), except for the AS and CE1 strains, whereas clade 3 displayed only four types of CAZyme profiles: CBM, CE, GH, and GT. The major CAZymes categories of LR6 were 13 genes of GH1 (β-glucosidase, EC 3.2.1.21) and 11 genes of GH13 (α-amylase, EC 3.2.1.1), which are important enzymes in the vagina environment that depolymerizes glycogen for producing lactic acid during fermentation process.

To link genetic diversity to probiotic features, mucosal adhesion genes associated with host interactions were analyzed (Figure 2B). Studies on *L. rhamnosus* GG, a well-documented reference strain, have shown several gene clusters associated with pathogens and vaginal colonization. spaCBA-strC1 is a proteinaceous pili coding operon previously observed in *L. rhamnosus GG* but not in GR-1 isolated from the vaginal tract. Comparably, those operons were absent from LR6 genomes also; alternatively, the spaFDE-strC2 operon was found. MBF with Pfam-MucBP domain repeats and the adhesion modulator MabA were also found upstream of the spaFDE-strC2 area in LR6. Putative lectin-like protein-coding genes (llp) are crucial factors for the adhesion ability of gastrointestinal and vaginal epithelial cells in *L. rhamnosus* GR-1. They are divided into three domains, and each region exhibits high sequence similarity with three genes from the LR6 genome. Taken together, the genes in LR6 that are linked to pilus-mediated mucosal adhesin have the potential for improved vaginal attachment and colonization as probiotics in the vagina.

### 3.3 Distribution of bacteriocin gene clusters

**FIGURE 3:**
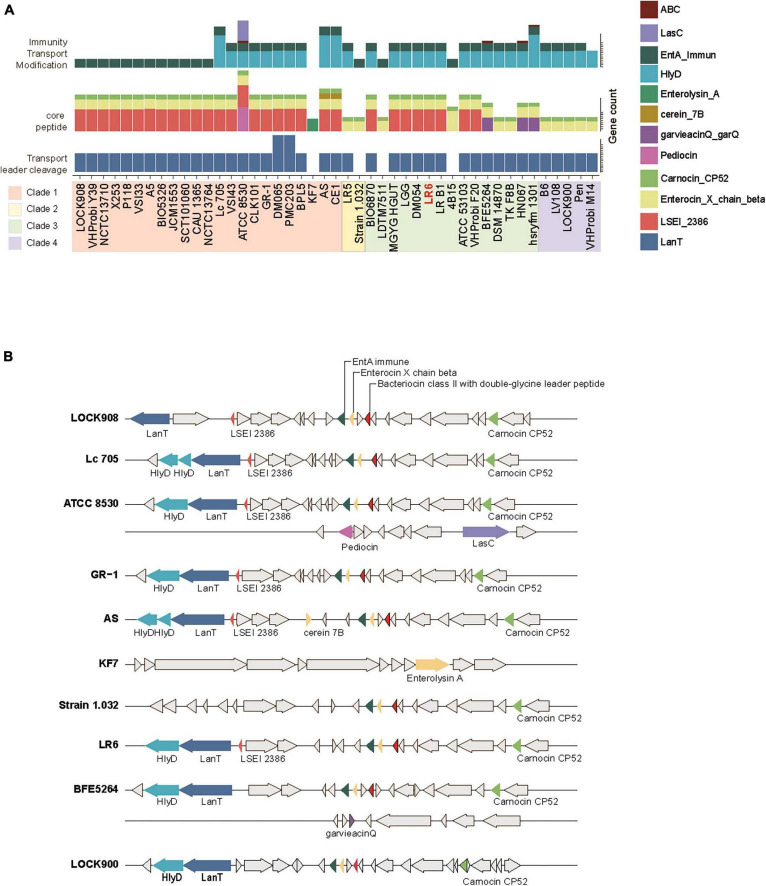
Distribution of biosynthetic gene cluster of bacteriocin genes of the 45 *L. rhamnosus* strains. **(A)** The number of gene count related to bacteriocin biosynthesis. Genes are colored based on their functions as indicate. Strains displayed with phylogenetic clade order. **(B)** Multiple sequence alignments of bacteriocin biosynthetic gene clusters among the representative strains from four phylogenetic clades. The color of gene arrow is filled in the same way as shown in panel **(A)**.

The BAGEL4 pipeline was used to determine the distribution of bacteriocin gene clusters among the 45 genomes of *L. rhamnosus*. Genes identified as open reading frames within bacteriocin biosynthetic gene clusters were categorized into three distinct functional groups: core peptide, transport/leader cleavage, and immunity/transport/modification. Considering that all strains exhibited Enterocin X chain beta and Carnocin_CP52 core peptides, with the exception of KF7, the most notable disparity between evolutionary clades was based on the presence of the LSEI_2386 domain from clades 1 or 3 (Figure 3A). Furthermore, it is worth noting that a particular core peptide, garvieacin Q, was detected only in certain strains within clade 3. Additionally, an absence of HlyD immune transporters in clade 1 was observed. However, these distinct genetic characteristics were not linked to the source of isolation. The distribution of the bacteriocin gene clusters, characterized by distinct patterns within each clade, is illustrated in Figure 3B. The prediction conducted for LR6 revealed the presence of HlyD and intA immunity transporters, in addition to the LanT exporter. Furthermore, three core peptide domains were identified: LSEI_2386, Enterocin, and Carnocin (Figure 3B). Taken together, the results indicate that LR6 possesses the genetic potential to synthesize class II bacteriocins through proper immune transporter and exporter genes, leading to an antimicrobial phenotype.

### 3.4 Antimicrobial activity of newly isolated LR6

**FIGURE 4:**
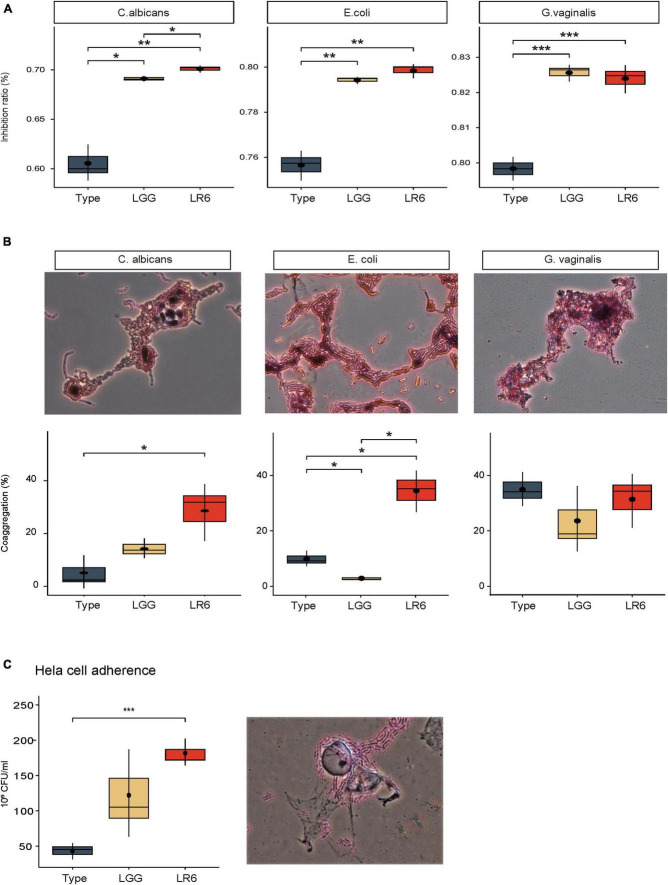
Antimicrobial and adhesive activities of *L. rhamnosus* LR6. **(A)** Comparison of antimicrobial activity of *L. rhamnosus strians*, ATCC7469, LGG, and LR6 against vaginal pathogens. **(B)** Evaluation of co-aggregation activity of three *L. rhamnosus* strains with vaginal pathogens 4 h after incubation. **(C)** HeLa cell adhesive activity of three strains of *L. rhamnosus* based on viable cell count method. The co-aggregation activity and HeLa cell adhesive capacity of LR6 were further supported by microscopic observations after Gram staining. *P < 0.05, **P < 0.01, and ***P < 0.001.

To investigate the probiotic characteristics associated with antimicrobial activity against vaginal pathogens, we evaluated their inhibitory effects against three common pathogens, *E. coli*, *C. albicans*, and *G. vaginalis*, which cause uropathogenic and vaginal infections. These effects were compared with those of the two control strains, *L. rhamnosus* type strains ATCC7469 and GG (Figure 4A). The inhibitory activity of LR6 against *C. albicans* was significantly higher than that against both the type and GG strains (*P*-value = 0.0085 and 0.019, respectively). In addition, LR6 exhibited a significantly higher percentage of inhibition against *E. coli* than the LR-type strain (*P*-value = 0.0025). Moreover, LR6 had a more significant inhibitory effect on *G. vaginalis* than the LR-type strain (*P*-value = 0.00099). The antimicrobial capability of these *L. rhamnosus* strains is well-documented to be primarily attributed to their hydrogen peroxide-producing characteristics. Therefore, we conducted an assessment of LR6’s hydrogen peroxide production capacity (Supplementary Figure 1). As a result, LR6 was observed to produce hydrogen peroxide, and it is anticipated that this contributes to its antimicrobial abilities, likely in conjunction with the bacteriocins identified in the genome analysis.

In addition to inhibiting the growth of pathogens in the vaginal environment, the co-aggregation ability is essential because it can facilitate the formation of a barrier that prevents the colonization of pathogenic bacteria. We measured the co-aggregation ability of the LR6 strain using the same strains (Figure 4B). LR6 exhibited co-aggregation values of 28.59% with *C. albicans*, which were significantly higher than those of the LR-type strain (*P*-value = 0.036). When tested against uropathogenic *E. coli*, LR6 showed a co-aggregation value of 33.56%, which was significantly higher than those of both the LR-type and LGG strains (*P*-value = 0.019, 0.017, respectively). In the case of *G. vaginalis*, LR6 demonstrated a co-aggregation ability of 36.33%, similar to that of both the LR-type strain and LGG, indicating substantial co-aggregation with *G. vaginalis*.

### 3.5 Adhesive activity of *L. rhamnosus* LR6 on HeLa cells

In addition to co-aggregation with pathogens, adhesion to vaginal epithelial cells is a critical step in establishing colonization. Based on a previous genetic investigation of adhesin factors, the adhesion ability of HeLa cells was investigated (Figure 4C). Our results demonstrated a significant difference in adhesive activity between *L. rhamnosus* LR6 and the type strain (*P*-value = 0.00079). The results demonstrated that LR6 showed significantly greater adhesion to HeLa cells, suggesting an enhanced capacity for interaction with vaginal epithelial cells. The adhesive capacity of LR6 was further supported by microscopic observations after Gram staining.

### 3.6 Organic acid production and acid tolerance

**FIGURE 5:**
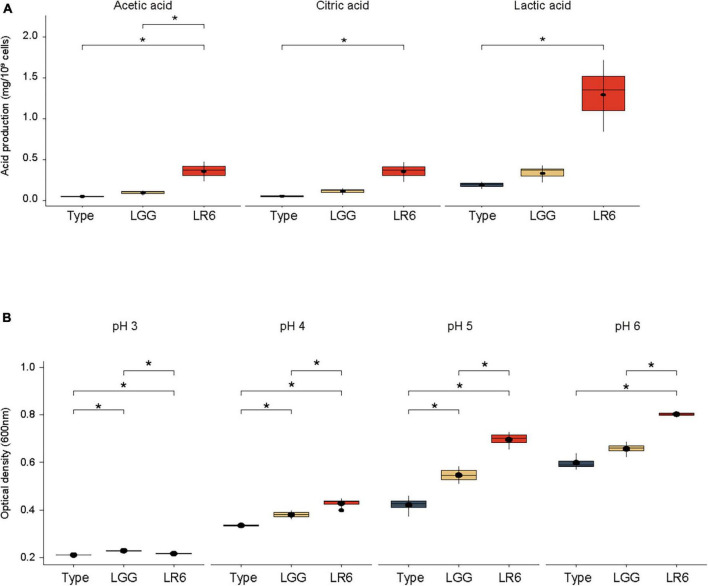
Organic acid production and acid tolerance of *L. rhamnosus* LR6. **(A)** Comparison of the concentrations of lactic acid, citric acid, and acetic acid produced by the three strains. **(B)** Comparison of the acidic tolerance under anaerobic conditions (pH ranging from 4 to 6). *P < 0.05, **P < 0.01, and ***P < 0.001.

In general, a healthy vaginal environment is strongly associated with organic acids produced by the dominant genus *Lactobacillus*. To evaluate the acidification ability of the vaginal environment, the concentrations of organic acids such as lactic acid, citric acid, and acetic acid produced by the three strains were measured (Figure 5A). When evaluating the organic acid production capabilities based on CFU quantities, it was noted that LR6 exhibited significantly elevated levels of lactic acid production than the ATCC7469 strain, measuring at 1.29 mg/10^9^ CFU (*P*-value = 0.043). Furthermore, LR6 showed significantly higher production of acetic acid (0.35 mg/10^9^ CFU). This value significantly surpassed the production levels of both the LR and LGG strains (*P*-value = 0.042 and *p* = 0.05, respectively). Regarding citric acid production, the LR6 strain had significantly better productivity than the LR-type strain (*P*-value = 0.043). To investigate the potential survival of the strains in a vagina-like environment, acidic tolerance under anaerobic conditions was also compared (Figure 5B). At pH 3, all three strains exhibited poor growth because of the harsh environmental conditions. However, under three different pH conditions (pH ranging from 4 to 6), particularly at pH 5, which represents the average acidity in the vagina, LR6 displayed a significantly higher growth rate than the ATCC7469 and LGG strains (*P*-value = 0.029 and 0.029, respectively).

### 3.7 Microbiome analysis

**FIGURE 6:**
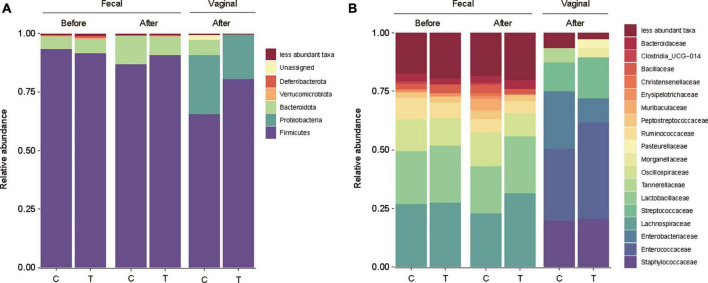
Microbial community analysis of fecal and vaginal microbiome of rat. **(A)** Taxonomy bar plot of fecal and vaginal microbiota before and after administration of *L. rhamnosus* LR6 over a two-week at phylum level, and **(B)** family level. Fecal samples were collected on days 0 and 14 and vaginal swabs were collected on day 14 (*n* = 12).

A rat model was used to assess the impact of *L. rhamnosus* LR6 administration. Rats were orally administered LR6 over a two-week period and fecal and vaginal samples were collected before and after administration. Analysis of the microbiome data revealed distinct shifts in the microbial composition following LR6 administration (Figure 6). At the phylum level, both the fecal and vaginal microbiota exhibited an increase in *Firmicutes* compared to the control samples. Notably, compared with that in the control sample, the family *Lactobacillaceae* demonstrated increased abundance in the fecal samples post-administration, indicating the potential colonization or transient presence of LR6 in the gut environment. In vaginal samples, *Enterobacteriaceae* notably decreased compared to that in the control samples, whereas *Enterococcaceae*, *Streptococcaceae*, and *Morganellaceae* increased. The observed changes in the microbiota composition suggest that orally administered *L. rhamnosus* LR6 traversed the gastrointestinal tract and influenced the vaginal microbiome.

## 4 Discussion

*Lactobacillus* is considered an essential genus in the vaginal environment that helps maintain the vaginal natural acidic pH, inhibits the growth of potentially harmful microbes, and stabilizes the microbial balance (Chee et al., 2020; Abbe and Mitchell, 2023; Liu et al., 2023). The identification of gene clusters within the genome involved in the synthesis of bacteriocins, as well as the existence of adhesive-associated genes in specific habitats, such as the vaginal tract, is of great importance in the exploration of new vaginal probiotics (Kankainen et al., 2009; Petrova et al., 2021; Wang et al., 2021). Therefore, in this study, the isolation and genomic analysis of *L. rhamnosus* LR6 from the vaginal microbiota of healthy females provided valuable insights into the potential of this strain as a new probiotic for vaginal health. Here, we report a new *L. rhamnosus* strain, LR6, sourced from the vaginas of 20 healthy Korean women, with various probiotic characteristics. Comparative genomic analysis revealed interesting features illustrating the suitability of LR6 for vaginal applications. In the context of vaginal health, the diverse origins of the 45 *L. rhamnosus* strains included in this study highlight the distinctive genomic characteristics of LR6. The prevalence of replication, recombination, and repair functions among cloud genes from diverse sources suggests that these strains adapted dynamically to their respective environments. Phylogenetic analysis classified LR6, along with other vaginal genomes, into four distinct phylogroups reflecting its evolutionary relationship within this specific niche (Figure 2A). The presence of carbohydrate-utilizing enzymes, as identified by CAZyme profiling, supports the vaginal environment specialization of LR6. Notably, LR6 possessed an abundant number of genes related to GH1, GH13, and α-amylase family, the corresponding enzymes being essential for glycogen depolymerization and lactic acid production (Nunn et al., 2020). Majority of vaginal glycogen is discharged by exfoliated epithelial cells and subsequently broken down by α-amylase, leading to its conversion into lactic acid by the predominant vaginal microbiota, primarily *Lactobacilli* (Nasioudis et al., 2015). This process is a significant source of nutrients and plays a role in maintaining an acidic vaginal environment (Hood Pishchany and Rakoff-Nahoum, 2019).

The genetic makeup of LR6 through mucosal adhesion highlights its potential as a vaginal probiotic. LR6 harbored genes associated with pilus-mediated mucosal adhesion, including the spaFDE-strC2 operon and putative lectin-like protein-coding genes (Figure 2B). The presence of MBF including Pfam-MucBP domain repeats and the adhesion modulator MabA was observed as well in the upstream region of the spaFDE-strC2 locus in LR6 (Vélez et al., 2010). Putative lectin-like protein-coding gene, llp, important factors for the adhesion ability of gastrointestinal and vaginal epithelial cells, also observed in the LR6 genome (Petrova et al., 2016). The presence of these genes may improve the ability of LR6 to attach to and colonize vaginal epithelial cells, thereby creating a large biological niche in the vaginal microbiome (Kankainen et al., 2009; Abbe and Mitchell, 2023).

The distribution of bacteriocin gene clusters across the genomes of *L. rhamnosus* gives a glimpse into the antimicrobial potential of LR6 (Turovskiy et al., 2009). The presence of a core peptide, transport/leader cleavage, and immunity/transport/modification genes suggests that this organism is capable of generating class II bacteriocins, which are among the most abundant and diverse secondary metabolites found in lactic acid bacteria (Zhang et al., 2023). This antimicrobial activity is consistent with LR6’s inhibitory effects against uropathogenic *E. coli* and two vaginal pathogens, *C. albicans* and *G. vaginalis*, as confirmed by *in vitro* analyses (Figure 4). In addition, the ability of LR6 to co-aggregate with vaginal pathogens and its tendency to adhere to HeLa cells indicate its potential to form a barrier against pathogens and prevent colonization (Reuben et al., 2019; Liu et al., 2023).

The vaginal environment is characterized by the dominance of lactic acid-producing *Lactobacillus* species, which form a stable microbiota. Therefore, the ability of vaginal probiotics to produce organic acids is of paramount importance (O’Hanlon et al., 2019). Growth in acidic milieu is a crucial attribute of these probiotic strains. The ability of LR6 to produce lactic acid, citric acid, and acetic acid suggests that it could potentially acidify the vaginal environment coupled with LR6’s robust growth under anaerobic conditions, especially at pH 5, to maintain a healthy vaginal pH (Figure 5).

In conclusion, *L. rhamnosus* LR6, isolated from the vagina of a healthy woman, is a promising candidate for a new vaginal probiotic. The genomic characteristics of LR6, including its antimicrobial activity, adhesion capabilities, and acidification potential, make it a potential probiotic for vaginal health maintenance. While these findings are promising, comprehensive clinical investigation are essential to validate the efficacy of LR6 and to determine the optimal delivery mechanisms and dosages for use in women’s health management. Further investigations, including clinical studies, are necessary to fully explore their benefits and potential applications in promoting vaginal well-being.

## Data availability statement

The datasets presented in this study can be found in online repositories. The names of the repository/repositories and accession number(s) can be found below: https://www.ncbi.nlm.nih.gov/, PRJNA1016041.

## Ethics statement

The studies involving humans were approved by the Institutional Review Board of the Konkuk University Medical Center (KUMC 2022-04-030). The studies were conducted in accordance with the local legislation and institutional requirements. The participants provided their written informed consent to participate in this study. The animal study was approved by Institutional Animal Care and Use Committee board in the CellBiotech (IACUC, approval No: CBT-2023-09). The study was conducted in accordance with the local legislation and institutional requirements.

## Author contributions

YC: Conceptualization, Data curation, Investigation, Methodology, Resources, Software, Supervision, Validation, Visualization, Writing – original draft. SK: Formal analysis, Writing – review and editing. DS: Formal analysis, Writing – review and editing. JL: Resources, Writing – review and editing. MC: Project administration, Writing – review and editing. SL: Formal analysis, Project administration, Resources, Supervision, Writing – review and editing.

## Footnotes

1
http://qiime2.org
